# Supplementary material for: Apparent symmetry rising induced by crystallization inhibition in ternary co-crystallization-driven self-assembly
Source: Nat Commun. 2023 Oct 14;14:6496. doi: 10.1038/s41467-023-42290-7 (PMC10576807; doi:10.1038/s41467-023-42290-7)
Supplement: Supplementary file 1 — Supplementary Information [file 41467_2023_42290_MOESM1_ESM.pdf]

# Supplementary Information

# Apparent symmetry rising induced by crystallization inhibition in ternary co-crystallization-driven self-assembly

Siyu Xie[1,2], Wenjia Sun[1], Xinhua Wan[1,2], Junliang Sun[1], Jie Zhang*[1,2]

[1] Beijing National Laboratory for Molecular Sciences, College of Chemistry and Molecular Engineering, Peking University, Beijing 100871, China.

[2] Key Laboratory of Polymer Chemistry and Physics of Ministry of Education, Peking University, Beijing 100871, China.

*Correspondence to: jz10@pku.edu.cn (J. Zhang)

## Table of Contents

1. Supplementary Notes ........ 2

1.1 Materials ........ 2

1.2 Measurements ........ 2

2. Supplementary Methods ........ 4

2.1 Synthetic procedures and characterization ........ 4

2.2 Le Bail refinement ........ 8

2.3 Calculation of $f_{PS}$ and $\varphi_{PEO\text{-}b\text{-}PS}$ ........ 10

2.4 Calculation of Hanson solubility parameters ........ 11

3. Supplementary Figures ........ 13

4. Reference ........ 21

# 1. Supplementary Notes

## 1.1 Materials

1-dodecanethiol (98%, Adamas), carbon disulfide (97%, Aladdin), 2-bromo-2-methylpropanoic acid (98%, Adamas), styrene (99%, Energy Chemical), 2,2'-azobis(2-methylpropionitrile) (AIBN, 99%, J&K Scientific), silicotungstic acid hydrate (STA, 97%, Aladdin), methoxypoly(ethylene glycol) 2000 ($PEO_{45}$, EP, TCI), 4-dimethylaminopyridine (DMAP, 99%, Aladdin), 1-Ethyl-3-(3-dimethylaminopropyl)carbodiimide hydrochloride (EDC · HCl, 98%, J&K Scientific), tetrahydrofuran (THF, HPLC, ThermoFisher), dichloromethane (DCM, HPLC, Concord Technology (Tianjin) Co.), ethanol (EtOH, HPLC, Concord Technology (Tianjin) Co.), acetone (AR, Beijing Tongguang Fine Chemicals Co.), methanol (HPLC, Concord Technology (Tianjin) Co.), *n*-hexane (HPLC, Concord Technology (Tianjin) Co.), petroleum ether (PE, CCER, Beijing Tongguang Fine Chemicals Co.), ethyl acetate (EA, CCER, Beijing Tongguang Fine Chemicals Co.), Toluene (AR, Beijing Tongguang Fine Chemicals Co.), hydrochloric acid (HCl, GR, Beijing Tongguang Fine Chemicals Co.), sodium chloride (AR, Beijing Tongguang Fine Chemicals Co.), tripotassium phosphate (AR, Beijing Tongguang Fine Chemicals Co.), sodium sulfate (AR, Beijing Tongguang Fine Chemicals Co.). AIBN was recrystallized three times from ethanol and dried under vacuum at room temperature.

## 1.2 Measurements

**Nuclear magnetic resonance (NMR)**: $^1$H NMR experiments were carried out on a Bruker ARX400 spectrometer at 25°C. The chemical shifts ($\delta$) in $^1$H NMR were reported in ppm relative to tetramethylsilane (TMS) as internal standard (0.0 ppm) or proton resonance resulting from incomplete deuteration of NMR solvent $CDCl_3$ (7.26 ppm). Coupling constants ($J$) are expressed in hertz.

**Gel permeation chromatography (GPC)**: The number-average molar masses ($M_n$), weight-average molar masses ($M_w$), and polydispersity indices ($Đ = M_w/M_n$) of the resultant polymers were estimated on a GPC instrument equipped with a Waters 515 HPLC pump and a Waters 2410 refractive-index detector. Three Waters Styragel columns with 10 μm bead size were connected in tandem. Their effective molar masses ranges were 100-10000 for Styragel HT2, 500-30000 for

Styragel HT3, and 5000-600000 for Styragel HT4, respectively. The pore sizes were 50, 100, and 1000 nm for Styragels HT2, HT3, and HT4, respectively. THF was used as the eluent at a flow rate of 1.0 mL/min at 35°C. The calibration curve was obtained against polystyrene standards.

**Transmission electron microscope (TEM)**: TEM, EDS, and selected area electron diffraction (SAED) images were obtained using Thermo Fisher Scientific (FEI) Tecnai F20 and JEOL JEM-2100 transmission electron microscope operating at 200 kV. The images were taken with Gatan Oneview IS camera. TEM samples were prepared by adding 10 μl solution to the thin pure carbon film coated Cu grids of 300 mesh and blotting away the excess solution with dust-free paper.

**Scanning electron microscope (SEM)**: SEM images were obtained on a Zeiss Merlin Compact field-emission scanning electron microscope operated at 10 KV. The samples were prepared by dipping a drop of solution onto hydrophilic cleaning silica wafers kept at 25°C and the solutions were absorbed by dust-free paper after 15 s. The wafers were stuck to the conductive adhesive.

**Atomic force microscope (AFM)**: AFM images were performed on a Bruker BioScope Resolve under ScanAsyst mode in the air. The ScanAsyst-Air probe was used and has one silicon tip on the nitride lever with a reflective Al back side. Scan rate 1 Hz, Peak Force Amplitude 150 nm, Peak Force Frequency 2 kHz. The AFM samples were prepared on silica wafers, the method of which is similar to the SEM ones.

**Grazing-incidence wide-angle X-ray scattering (GIWAXS)**: The GIWAXS image was performed on SAXS Lab Ganesha calibrated by $LaB_6$ and silver behenate. Grazing-incident angle 0.15°, sample to detector 100 mm, measure time 600 s. The GIWAXS samples were prepared on silica wafers, the method of which is similar to the SEM ones.

**Powder X-ray diffraction (PXRD)**: The PXRD of STA/PEO was measured by PANalytical X'Pert Pro with Cu Kα radiation at room temperature in the air. Tension 40 kV, Current 40 mA, $2\theta$ angle 4–50°, Scan speed 0.07°/s.

## 2. Supplementary Methods

### 2.1 Synthetic procedures and characterization

**Synthesis of 2-dodecylsulfanylthiocarbonylsulfanyl-2-methyl-propanoic acid (DMP)**

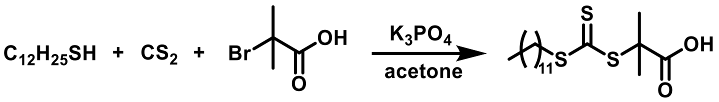


Dodecane thiol (4.10 g, 20.26 mmol) was added dropwise to a suspension of $K_3PO_4 \cdot 3H_2O$ (5.27 g, 19.76 mmol) in acetone (50 mL), and stirred for 30 minutes. Carbon disulfide (4.10 g, 53.85 mmol) was added and stirred for 10 minutes. 2-bromo-2-methylpropionic acid (3.10 g, 18.56 mmol) was added and stirred overnight at room temperature followed by concentration under reduced pressure. 1 M HCl (200 mL) was added and extracted with dichloromethane (DCM, 2 × 200 mL). The organic extracts were collected, washed with water (200 mL) and saturated NaCl solution (200 mL) respectively, then dried with anhydrous $Na_2SO_4$. The solvent was removed under reduced pressure and the residue was purified by column chromatography on silica with the eluent (petroleum ether (PE) /ethyl acetate (EA) = 4/1, *V*/*V*). The crude product was recrystallized in PE to obtain 2.0 g of yellow powder with a yield of 30%.

$^1$H NMR (400 MHz, $CDCl_3$, $\delta$, ppm): 3.28 (t, $J$ = 7.4 Hz, 2H, –SC$\underline{H}_2$–); 1.73 (s, 6H, –SC(C$\underline{H}_3$)$_2$–); 1.65–1.58 (m, 2H, –SCH$_2$C$\underline{H}_2$–); 1.39–1.31 (m, 2H, –SCH$_2$CH$_2$C$\underline{H}_2$–); 1.25 (m, 16H, CH$_3$(C$\underline{H}_2$)$_8$–); 0.88 (t, $J$ = 4.3 Hz, 3H, –CH$_2$C$\underline{H}_3$).

$^1$H NMR spectrum (400 MHz) of DMP in Chloroform-*d* at 25°C.

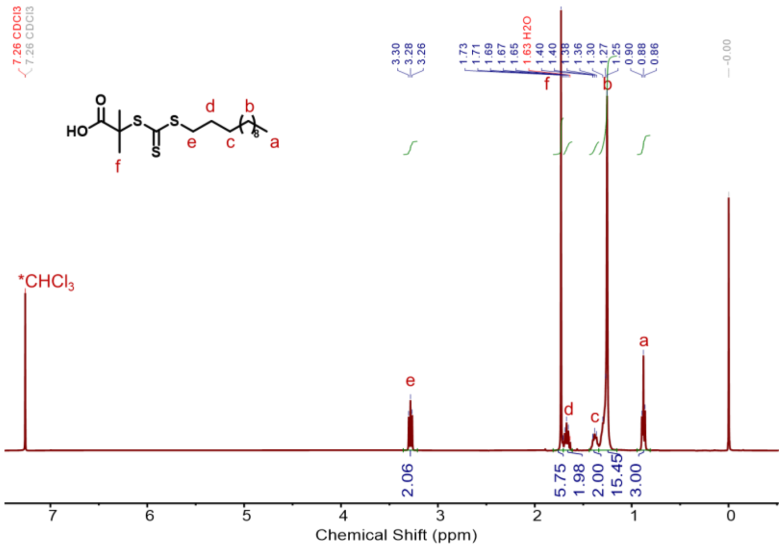

**Synthesis of polyethylene glycol 2-dodecylsulfanylthiocarbonylsulfanyl-2-methyl-propanate ($PEO_{45}$-DMP)**

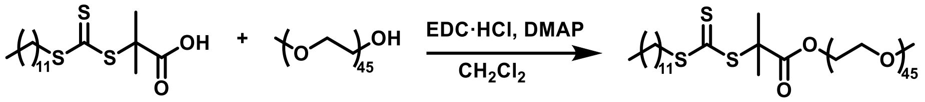


Polyethylene oxide monomethyl ether 2000 ($PEO_{45}$, 2.0 g, 1 mmol), 1-(3-dimethylaminopropyl)-3-ethylcarbodiimide hydrochloride (EDC·HCl, 1.2 g, 6 mmol), and 4-dimethylaminopyridine (DMAP, 74 mg, 0.6 mmol) were dissolved in DCM (100 mL), and the DCM solution of DMP (20 mL, 0.73 g, 2 mmol) was added dropwise with stirring at 0°C under nitrogen. The temperature was slowly raised to room temperature after dropping and kept stirring for a week. The mixture was concentrated under reduced pressure and purified by column chromatography on silica with the eluent (DCM/methanol = 20/1, *V*/*V*). The obtained product was dissolved in tetrahydrofuran (THF) and precipitated in diethyl ether followed by filtration and drying in a vacuum to give a yellow powder (2.3 g, 98%).

$^1$H NMR (400 MHz, $CDCl_3$, *δ*, ppm): 4.23 (t, 2H, –COOC$\underline{H}_2$–), 3.82–3.41 (m, 178H, –$CH_2C\underline{H}_2O(C\underline{H}_2C\underline{H}_2O)_{44}CH_3$), 3.35 (s, 3H, –OC$\underline{H}_3$), 3.24 (t, *J* = 7.4 Hz, 2H, –SC$\underline{H}_2$–), 1.67 (s, 6H, –SC(C$\underline{H}_3$)$_2$–), 1.65–1.58 (m, 2H, –$SCH_2C\underline{H}_2$–), 1.39–1.31 (m, 2H, –$SCH_2CH_2C\underline{H}_2$–), 1.23 (m, 16H, $CH_3(C\underline{H}_2)_8$–), 0.85 (t, *J* = 4.3 Hz, 3H, –$CH_2C\underline{H}_3$).

$^1$H NMR spectrum (400 MHz) of $PEO_{45}$-DMP in Chloroform-*d* at 25°C.

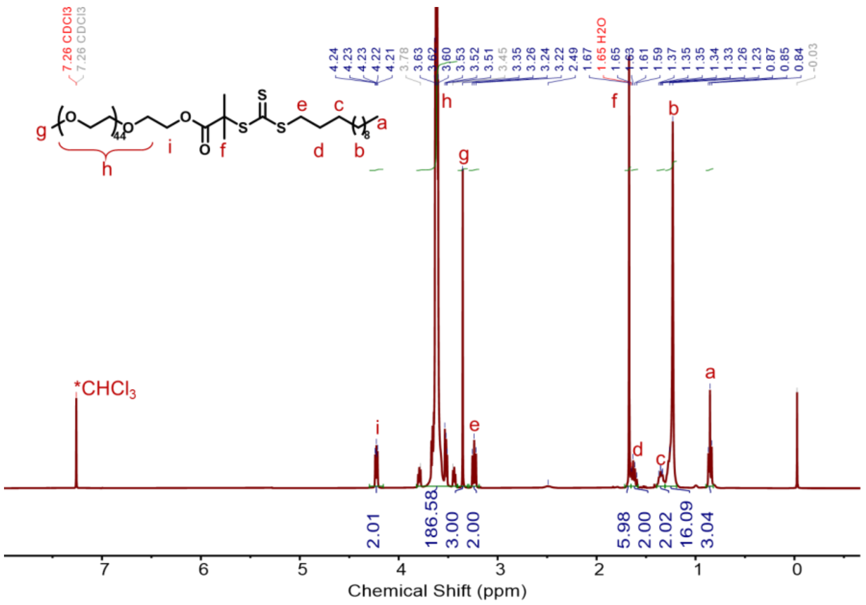

**Synthesis of the block copolymer polyethylene oxide-*b*-polystyrene (PEO-*b*-PS)**

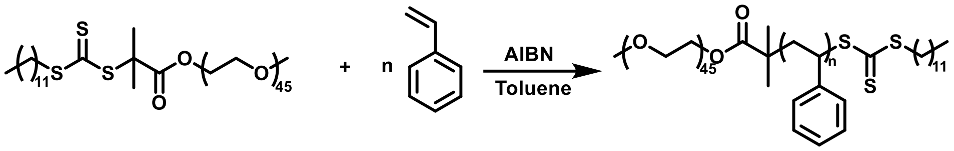


A series of block copolymers with different block ratios were obtained via reversible addition-fragmentation chain transfer (RAFT) polymerization in solution. A typical process was presented as follows: The macromolecular chain transfer agent $PEO_{45}$-DMP (117 mg, 0.05 mmol), styrene (260.37 mg, 2.5 mmol), initiator AIBN (1.64 mg, 0.01 mmol), and dry toluene (0.7 mL) were added to a Schlenk flask and stirred until dissolved. The system was degassed by freeze–pump–thaw three times, and the RAFT polymerization was performed at 90°C for 24 h. The reaction was quenched in liquid nitrogen. The reaction mixture was diluted and precipitated in a large amount of *n*-hexane, and a yellow solid (170 mg, 45%) was obtained. The dispersity *Đ* was 1.08 by GPC of THF eluent. The obtained polymers were further purified by preparative GPC of chloroform eluent to obtain narrowly distributed polymers with *Đ* of 1.04.

$^1$H NMR (400 MHz, $CDCl_3$, $\delta$, ppm): 7.22–6.92 (m, 48H, Ar–$\underline{H}$), 6.92–6.26 (m, 32H, Ar–$\underline{H}$), 3.89–3.43 (m, 180H, –$C\underline{H}_2C\underline{H}_2O$–), 3.38 (s, 3H, –$OC\underline{H}_3$), 2.43–0.63 (m, 79H, –$C\underline{H}_2C\underline{H}$Ar–, –OOCC($C\underline{H}_3$)$_2$–, –$C_{12}\underline{H}_{25}$).

**Supplementary Table 1.** Synthesis of $PEO_{45}$-*b*-$PS_n$ by RAFT.

| Sample [a] | Feeding Ratio | | | $M_n$ [b] (kDa) | *Đ* [b] | Yield [c] (%) |
|---|---|---|---|---|---|---|
| | St | PEO-DMP | AIBN | | | |
| $PEO_{45}$-*b*-$PS_3$ | 62.5 | 5 | 1 | 3.7 | 1.03 | 29 |
| $PEO_{45}$-*b*-$PS_9$ | 125 | 5 | 1 | 4.4 | 1.03 | 54 |
| $PEO_{45}$-*b*-$PS_{16}$ | 250 | 5 | 1 | 5.4 | 1.04 | 45 |
| $PEO_{45}$-*b*-$PS_{33}$ | 500 | 5 | 1 | 7.0 | 1.04 | 65 |
| $PEO_{45}$-*b*-$PS_{47}$ | 750 | 5 | 1 | 9.0 | 1.04 | 51 |
| $PEO_{45}$-*b*-$PS_{82}$ | 1000 | 5 | 1 | 14.3 | 1.06 | 53 |

[a] The degrees of polymerization (*DP*s) of the blocks were determined by $^1$H NMR. [b] $M_n$ and *Đ* were obtained by GPC in THF. [c] Yield was calculated before purification by preparative GPC.

**Preparation of silicotungstic acid hexahydrate (STA)**

A large amount of commercial reagent silicotungstic acid hydrate ($H_4SiW_{12}O_{40} \cdot nH_2O$) was dissolved in deionized water to saturation and stirred for 10 minutes. The mixture was filtrated followed by concentration via heating at 100°C until the solution volume to 10 mL. A large number of colorless crystals were obtained at room temperature. The crystals were filtrated and ground into powder in an agate mortar, then heated at 100°C until no vapor was generated and transferred to a vacuum oven for further drying. After the crystal water is sufficiently removed, a white solid powder whose chemical structure was determined by thermogravimetric analysis (TGA) to be $H_4SiW_{12}O_{40} \cdot 6H_2O$ was obtained.

FT-IR spectrum (KBr plate, wavenumber, $cm^{-1}$): 1018, 980, 925, 892, 879, 782, 540, 479, 418.

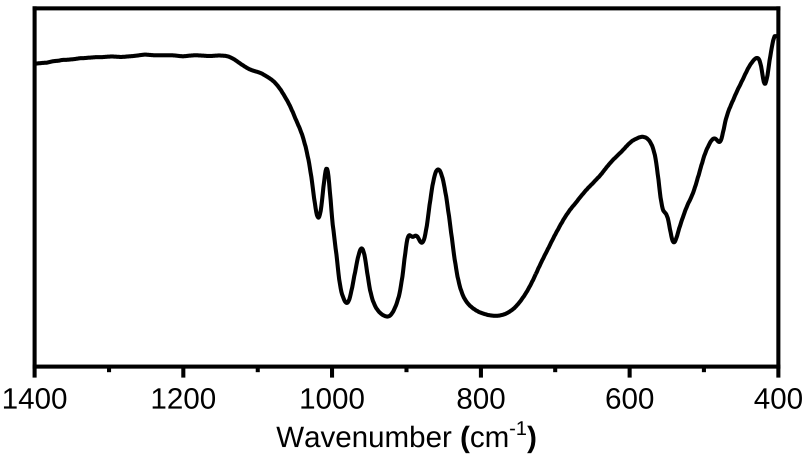


TGA spectrum of STA ramp 10°C/min to 800°C under air flowing at 100 mL/min.

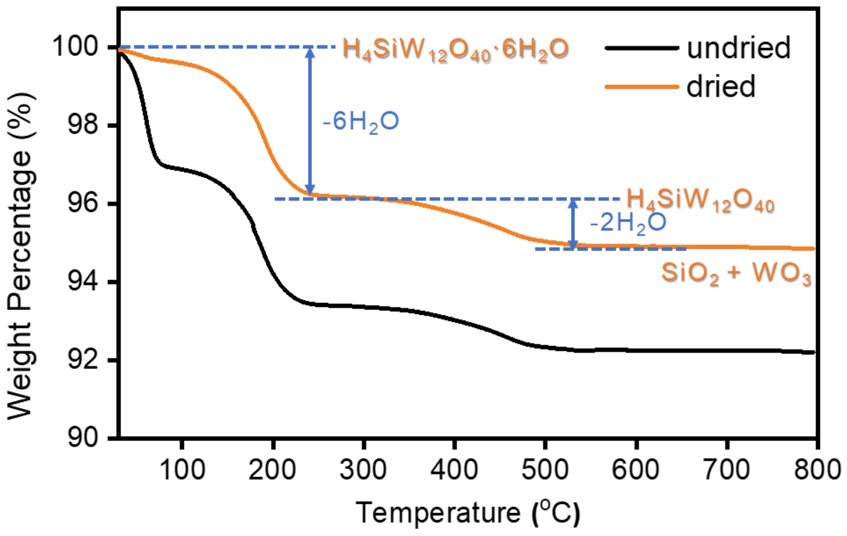

**A typical experimental procedure of kinetic-controlled CCDSA:** A series of solutions of silicotungstic acid (3 mmol/L) were prepared with THF containing 1 vol.% $H_2O$ and cooled at 0°C. The polymer mixed solution of n($PEO_{45}$)/n($PEO_{45}$-b-$PS_{16}$) ~ 1/1 at a total concentration of 2.50 mmol/L in THF/DCM ~3/1 (V/V) were prepared and cooled at 0°C. A mixture of 100 μL polymer solution was slowly added with 50 μL STA solution at 0°C, stand for 24 h in the refrigerator, and then the self-assembled solution containing the triangular nanoplates was obtained without further treatment.

**Experimental procedure of thermodynamic-controlled CCDSA:** After the triangular nanoplates were obtained through the above experimental procedures, the self-assembled solution containing the triangular nanoplates was sealed and heated to 80°C for 10 minutes, gradually cooled to room temperature and stand for 1~ 3 days.

## 2.2 Le Bail refinement

Jana 2006[1] was used for Le Bail fitting, and the process is as follows:

**Pattern matching**

Import data collected by powder X-ray diffraction (PXRD); Select "Various CW formats→PANalytical XRDML→Bragg-Brentanno method-Fixed Divergence Slit"; Enter cell parameters and select "Kalpha1/Kalpha2 doublet→X-rays Cu".

"Edit Powder parameters": In "cell" page select refinement of a, b, and c; In "Profile" page select "Gaussian" as peak-shape function with a cutoff to 12*FWHM and activate refinement of "GW"; In "Corrections" page activate refinement of "shift" and select "Legendre polynomials" with 5 terms for background calculation.

"Edit Refinement commands": In "Basic" page set 10 refinement cycles.

"Run Refine": The refinement will stop if max (change/s.u.) < 0.05.

"Show powder profile": The peaks are systematically broader, indicating that Lorentzian broadening is to be combined with the Gaussian profiles.

"Edit Powder parameters": In "Profile" page select "Pseudo-Voigt" and activate refinement of "LY"

"Run Refine"

"Edit Powder parameters": In "Profile" page activate refinement of "LX"

“Run Refine”: The peaks have no asymmetry problem, so activation and refinement of parameters “X+” and “X-” are not required.

**Space group determination**

“Tolerances for crystal system recognition”: Maximal deviation for cell lengths in 0.02 Å; Maximal deviation for cell angles in 0.2°.

“Select Laue symmetry”: Select Orthorhombic mmm Laue symmetry.

“Select cell centering”: Select C centering (because it has low $R_p$ factors and high extinction).

“Select space group”: Select $C222_1$ (because it has low $R_p$ factors and high extinction).

“Final step of the space group test”: Select accept the space group transformed into the original cell.

Run Le Bail refinement without reflections absent due to the new symmetry.

**Supplementary Table 2.** Summary of the Le Bail refinement results of STA/PEO.

| Name | STA/PEO |
|---|---|
| Crystal system | Orthorhombic |
| Unit cell | a = 16.420(6)Å<br>b = 27.297(7)Å<br>c = 26.668(8)Å<br>α = β = γ = 90° |
| Space group | $C222_1$ |
| Volume/Å$^3$ | 11954.15 |
| Z [a] | 4 |
| X-ray source | Cu Kα |
| Wavelength/Å$^3$ | 1.5405 |
| $2\theta$ | 5–40 |

| | |
|---|---|
| Number of reflections | 2543 |
| Number of parameters | 13 |
| Number of data points | 2096 |
| Refinement method | Le Bail refinement |
| $R_p$ | 1.45% |
| $R_{wp}$ | 2.32% |
| GoF | 2.29 |

[a] Z represents the number of STA in one unit cell.

## 2.3 Calculation of $f_{PS}$ and $\varphi_{PEO\text{-}b\text{-}PS}$

The volume fraction ($f_{PS}$) of the PS block is calculated as follows:

$$f_{PS} = \frac{\varphi_{PS-b-PEO} \cdot M_{n\ PS}/\rho_{PS}}{\varphi_{PS-b-PEO} \cdot M_{n\ PS}/\rho_{PS} + N_A \cdot N \cdot V_{STA/PEO}} \quad (1)$$

The molar ratio of block copolymers to total polymers ($\varphi_{PEO\text{-}b\text{-}PS}$) is calculated as follows:

$$\varphi_{PS-b-PEO} = \frac{n_{PS-b-PEO}}{n_{PS-b-PEO} + n_{PEO}} \quad (2)$$

$M_{n\ PS}$ represents the number-average molecular weight of PS block; $\rho_{PS}$ represents the density of PS, which is about 1.052 g/cm$^3$; $V_{STA/PEO}$ represents the unit cell volume of STA/PEO shown in **Supplementary Table 2**; $N_A$ stands for Avogadro constant, $6.02\times10^{23}$ mol$^{-1}$; The constant $N$ was calculated as the degree of polymerization of PEO divided by the number of repeat unit EO contained in a unit cell, which is 45/16.

The $f_{PS}$ of amorphous worm-like micelles was also calculated in the same way, since the constant $N$ is hard to estimate due to the uncertainty of the combination ratio of PEO and STA. As the true $N$ in the case of the amorphous structure is small than that of the crystalline structure, the real $f_{PS}$ should be larger than the calculated value. The calculated $f_{PS}$ is also of reference significance, reflecting that the ternary system cannot form crystalline assemblies by crystallization-driven self-assembly in the case of calculated $f_{PS}$.

## 2.4 Calculation of Hanson solubility parameters

The term Hanson solubility parameter (HSP)[2] is an important quantity for predicting solubility relations and is defined as follows:

$$\boldsymbol{\delta}^2 = \boldsymbol{\delta}^2{}_{\mathrm{D}} + \boldsymbol{\delta}^2{}_{\mathrm{P}} + \boldsymbol{\delta}^2{}_{\mathrm{H}} \tag{3}$$

HSP is a three-dimensional vector $\boldsymbol{\delta}\ (\boldsymbol{\delta}_{\mathrm{D}}, \boldsymbol{\delta}_{\mathrm{P}}, \boldsymbol{\delta}_{\mathrm{H}})$, which can be well characterized by just three parameters $\boldsymbol{\delta}_{\mathrm{D}}$ for dispersion (van der Waals), $\boldsymbol{\delta}_{\mathrm{P}}$ for polarity (related to dipole moment), and $\boldsymbol{\delta}_{\mathrm{H}}$ for hydrogen bonding. The equivalent HSP for mixed solvent is the weighted vector sum of each component:

$$\boldsymbol{\delta} = \sum_i x_i \boldsymbol{\delta}_i \tag{4}$$

The compatibility between two materials can be represented as the relative energy density (RED), which is defined as:

$$\mathrm{RED} = \mathrm{R_a}/\mathrm{R_0} \tag{5}$$

Where $R_a$ is the distance of HSP for two materials and is calculated as:

$$\mathrm{R_a} = \sqrt{4(\boldsymbol{\delta}_{\mathrm{D2}} - \boldsymbol{\delta}_{\mathrm{D1}})^2 + (\boldsymbol{\delta}_{\mathrm{P2}} - \boldsymbol{\delta}_{\mathrm{P1}})^2 + (\boldsymbol{\delta}_{\mathrm{H2}} - \boldsymbol{\delta}_{\mathrm{H1}})^2} \tag{6}$$

$R_0$ is the radius of the solute with which the other material must be compatible.

RED less than 1 indicates high affinity, while RED more than 1 indicates low affinities. RED equal or close to 1 indicates a critical state.

The HSPs of THF, EtOH, DCM, $H_2O$, and PS are looked up and listed in **Supplementary Table 3**, the $R_0$ of PS is 12.7, and the solubility of PS in mixed solvents can be calculated and displayed in **Supplementary Table 3**.

**Supplementary Table 3.** Hanson solubility parameters of mixed solvents and the solubility of PS.

| Solvent [a] | $\delta_D$ | $\delta_P$ | $\delta_H$ | $R_a$ | RED |
|---|---|---|---|---|---|
| PS | 21.3 | 5.8 | 4.3 | - | - |
| THF | 16.8 | 5.7 | 8 | 9.731 | 0.766 |
| EtOH | 15.8 | 8.8 | 19.4 | 18.921 | 1.490 |
| DCM | 18.2 | 6.3 | 6.1 | 6.475 | 0.510 |
| $H_2O$ | 15.5 | 16.0 | 42.3 | 41.020 | 3.230 |
| $THF_6/DCM_0/H_2O_{0.02}$ | 16.796 | 5.734 | 8.114 | 9.783 | 0.770 |
| $THF_5/DCM_1/H_2O_{0.02}$ | 17.028 | 5.834 | 7.798 | 9.232 | 0.727 |
| $THF_4/DCM_2/H_2O_{0.02}$ | 17.261 | 5.934 | 7.483 | 8.684 | 0.684 |
| $THF_5/DCM_1/H_2O_{0.04}$ | 17.023 | 5.868 | 7.913 | 9.285 | 0.731 |
| $THF_5/DCM_1/H_2O_{0.01}$ | 17.031 | 5.817 | 7.741 | 9.206 | 0.725 |
| $THF_5/DCM_1/H_2O_{0.005}$ | 17.032 | 5.808 | 7.712 | 9.193 | 0.724 |
| $EtOH_4/DCM_4/H_2O_{0.02}$ | 16.996 | 7.571 | 12.824 | 12.242 | 0.964 |
| $EtOH_6/DCM_0/H_2O_{0.02}$ | 15.799 | 8.824 | 19.476 | 18.987 | 1.495 |
| $EtOH_5/DCM_1/H_2O_{0.02}$ | 16.198 | 8.409 | 17.267 | 16.706 | 1.315 |
| $EtOH_4/DCM_2/H_2O_{0.02}$ | 16.596 | 7.993 | 15.057 | 14.458 | 1.138 |
| $EtOH_5/DCM_1/H_2O_{0.04}$ | 16.195 | 8.434 | 17.350 | 16.777 | 1.321 |
| $EtOH_5/DCM_1/H_2O_{0.01}$ | 16.199 | 8.396 | 17.225 | 16.670 | 1.313 |
| $EtOH_5/DCM_1/H_2O_{0.005}$ | 16.199 | 8.390 | 17.204 | 16.652 | 1.311 |

[a] The subscripts denote the volume equivalent of the solvents.

## 3. Supplementary Figures

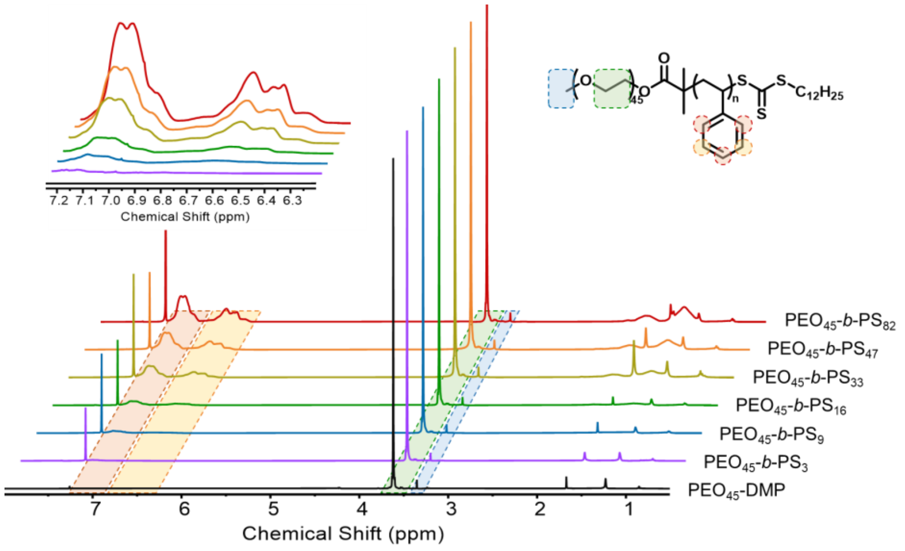


**Supplementary Fig. 1 | $^1$H NMR spectra of a set of PEO-*b*-PS with different molar weights.** 400 MHz, in chloroform-*d* at 25°C.

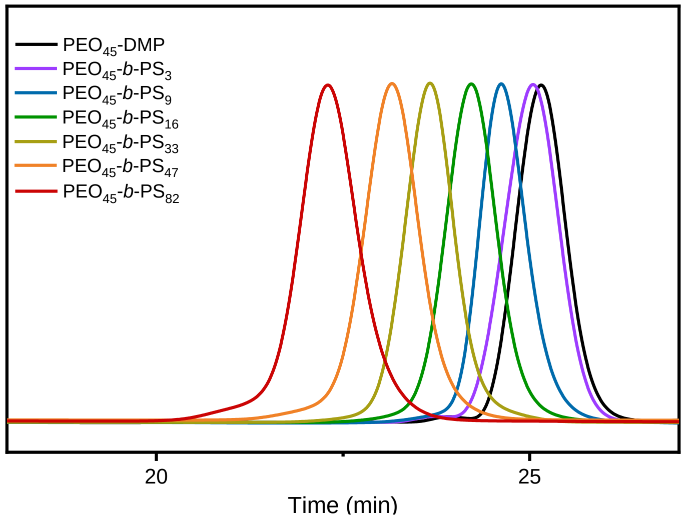


**Supplementary Fig. 2 | GPC curves of a set of PEO-*b*-PS with different molar weights.** The subscripts denote the degrees of polymerization of PS, which were calculated based on the $^1$H NMR.

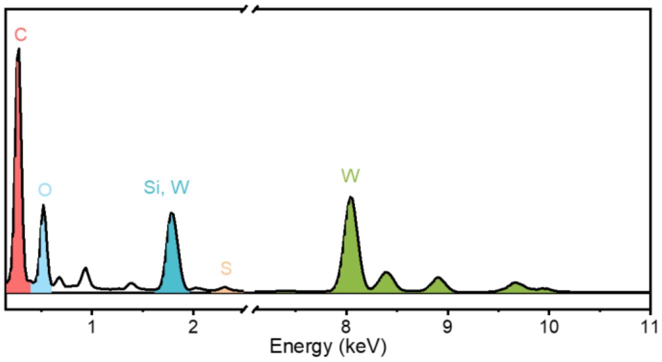


**Supplementary Fig. 3 | A cumulative energy-dispersive X-ray spectrum (EDS) of triangular nanoplates.**

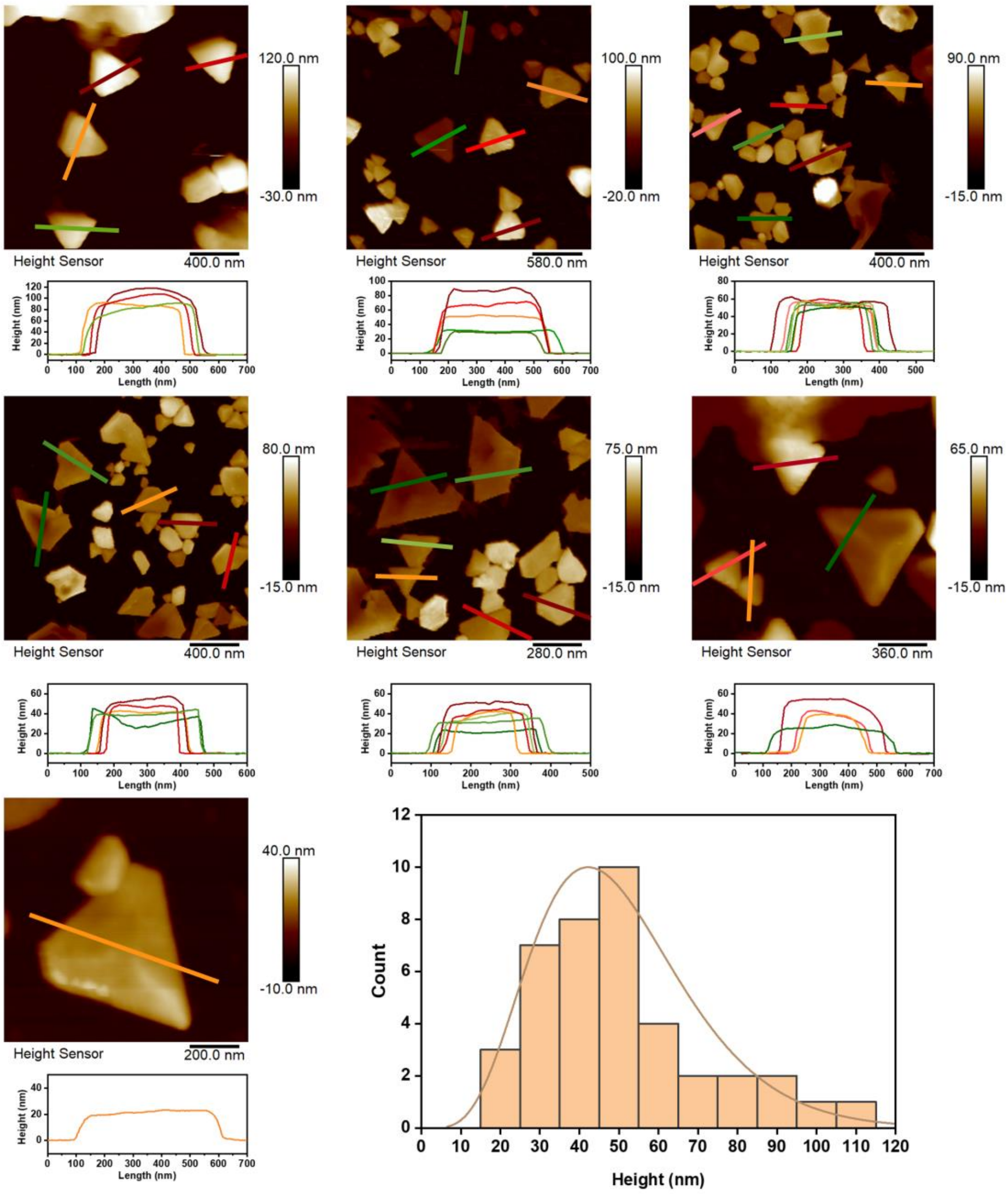


**Supplementary Fig. 4 | Height analysis of STA/PEO/PEO-*b*-PS triangular nanoplates.** AFM images, corresponding height profiles along the lines, and statistical analyses of the height distribution of 40 nanoplates.

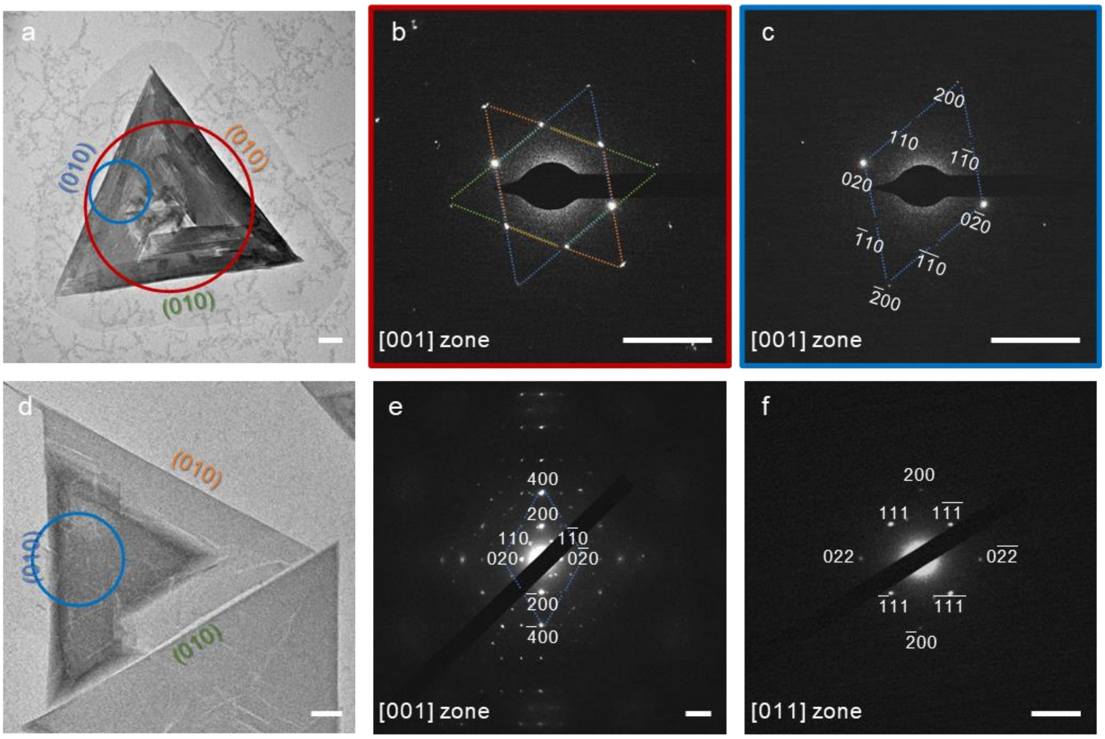


**Supplementary Fig. 5 | Analysis of crystal structure of triangular nanoplates. a** TEM image of the triangular nanoplate and corresponding SAED patterns taken from overall (**b**) and local (**c**) selected regions along the [001] directions. **d** TEM image and corresponding SAED patterns along the [001] (**e**) and [011] (**f**) directions. The scale bars are 200 nm for (**a**, **d**) and 1 nm$^{-1}$ for (**b**, **c**, **e**, **f**).

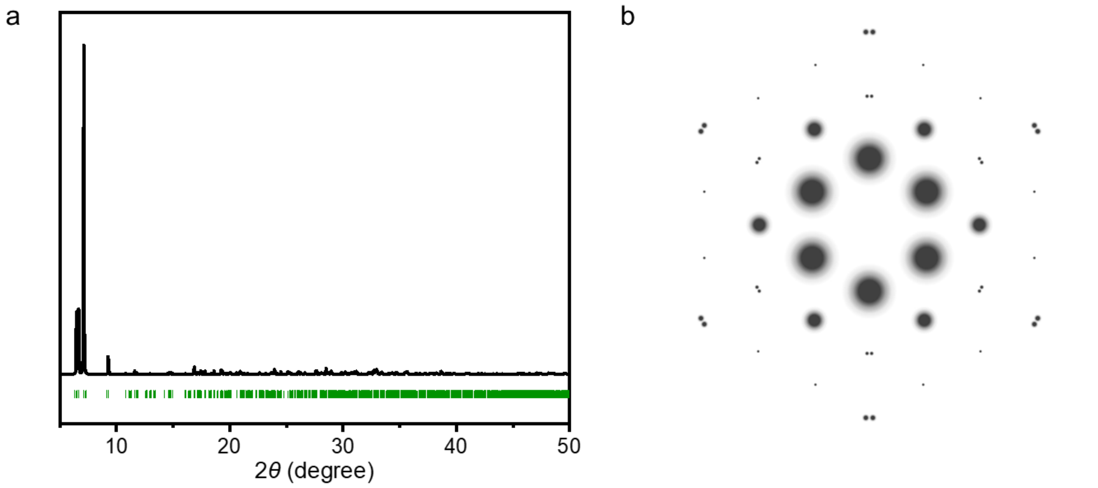


**Supplementary Fig. 6 | Simulation of crystal structure of triangular nanoplates.** The simulated XRD profile (**a**) and SAED pattern (**b**) along the [001] direction for the STA/PEO crystal structure.

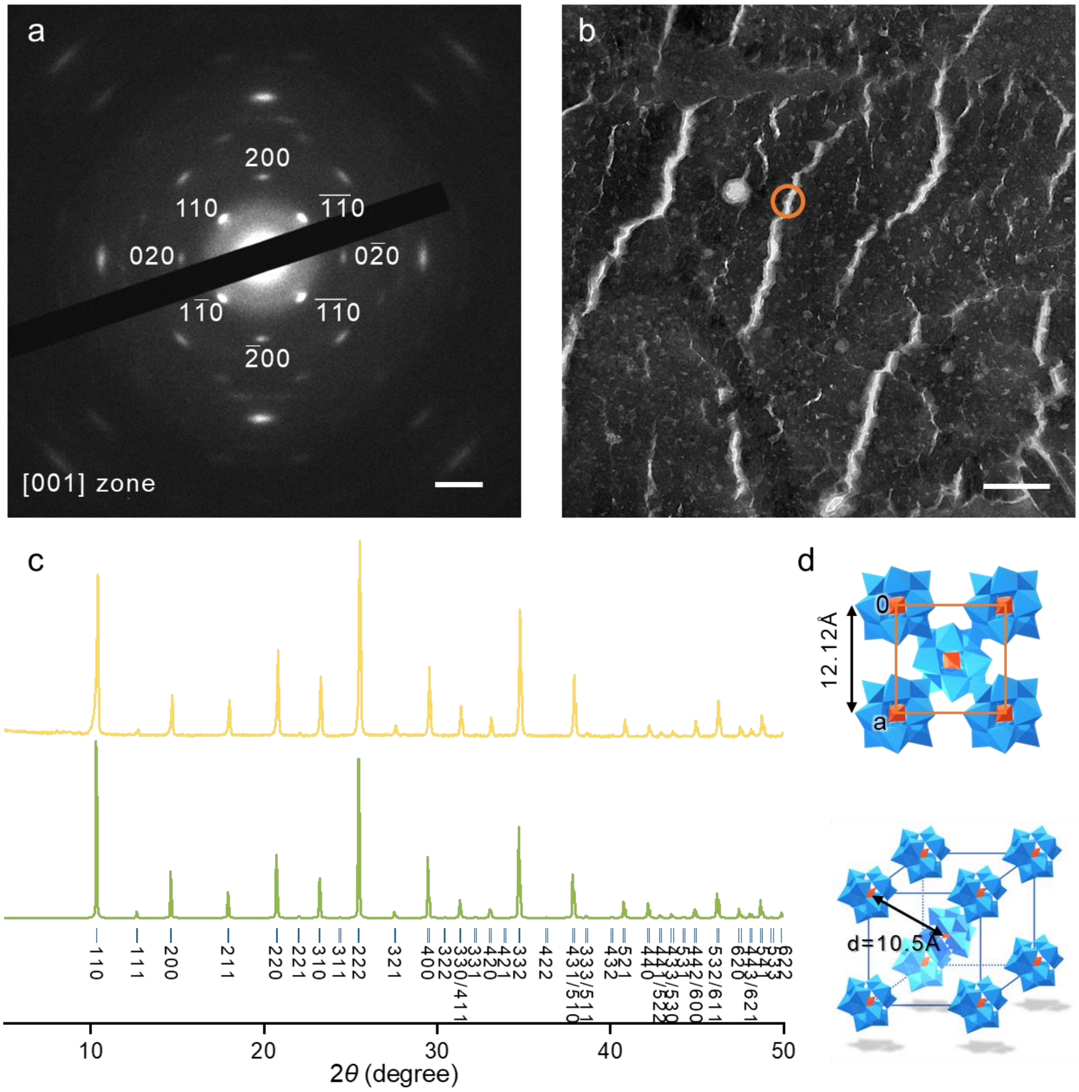


**Supplementary Fig. 7 | Analysis of crystal structure of STA.** SAED patterns (**a**) and TEM image (**b**) of STA by volatilizing the solution. **c** PXRD patterns of $H_4SiW_{12}O_{40}{\cdot}6H_2O$. PXRD profiles of experimental data (yellow curve), calculated simulated data (green curve), and Bragg position (blue curve). **d** Schematic representations of STA with $Pn\bar{3}m$ space group. The scale bars are 200 nm for (**a**) and 1 nm$^{-1}$ for (**b**).

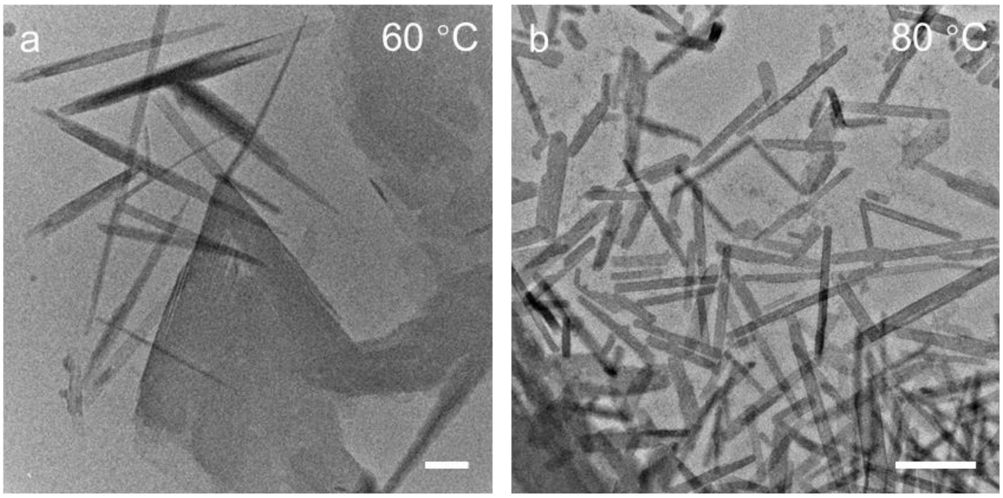


**Supplementary Fig. 8 | Effect of annealing temperature on the final morphology.** TEM images of incomplete transformation of the triangular nanoplates and nanoribbons at an annealing temperature of 60°C (**a**) and 80°C (**b**). All scale bars are fixed to 200 nm.

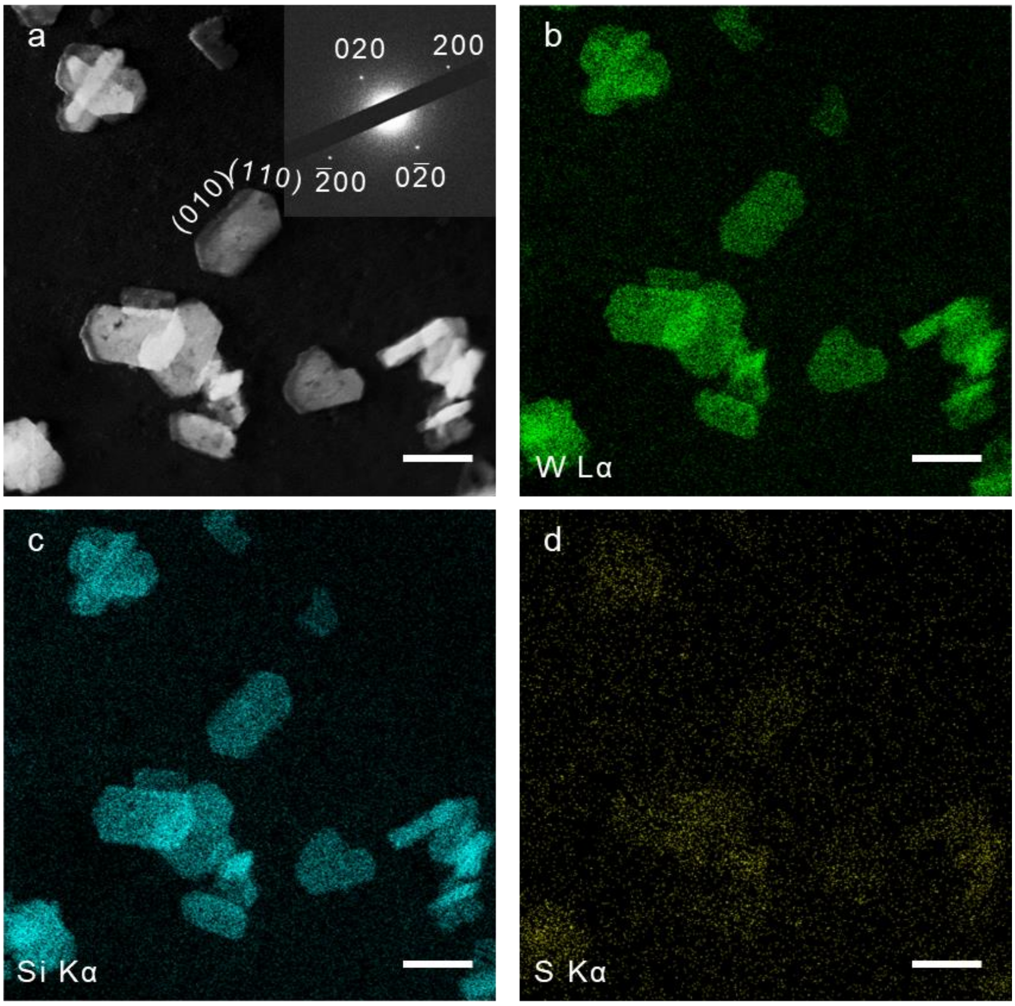


**Supplementary Fig. 9 | Morphological characterization and elemental analysis of nanosheets.** **a** HAADF STEM image of nanosheets. Corresponding EDS elemental distribution maps of W (**b**), Si (**c**), and S (**d**). All scale bars are fixed to 200 nm.

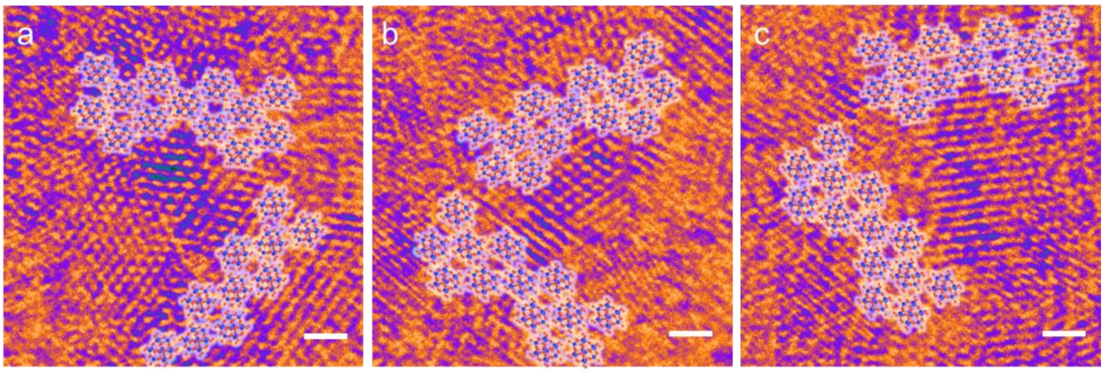


**Supplementary Fig. 10 | Pseudo-color high-resolution TEM images with schematical representations of STA.** The images a, b and c were collected from three different individual samples, which represent the same orientation attachment of STA. All scale bars are fixed to 1 nm.

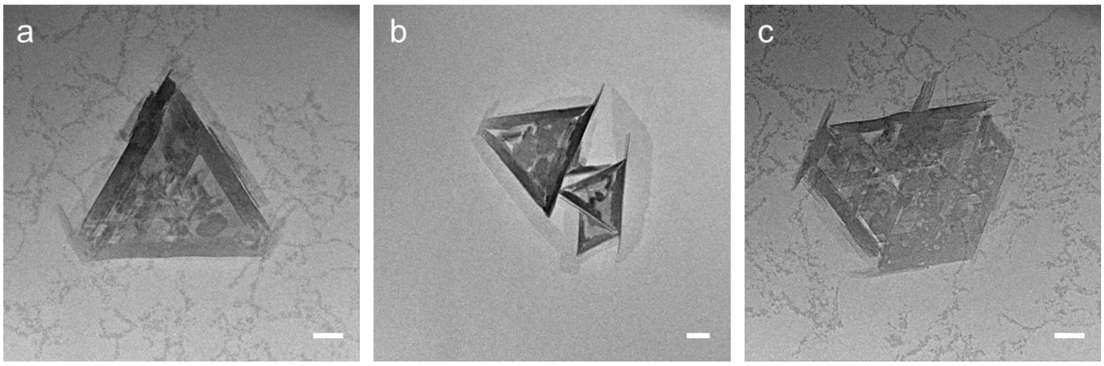


**Supplementary Fig. 11 | TEM images of imperfect triangular nanoplates showing the oriented substructures.** a,b) defective nanoplates with oriented triangle frames; c) distorted hexagonal multiply twinned particle. All scale bars are fixed to 200 nm.

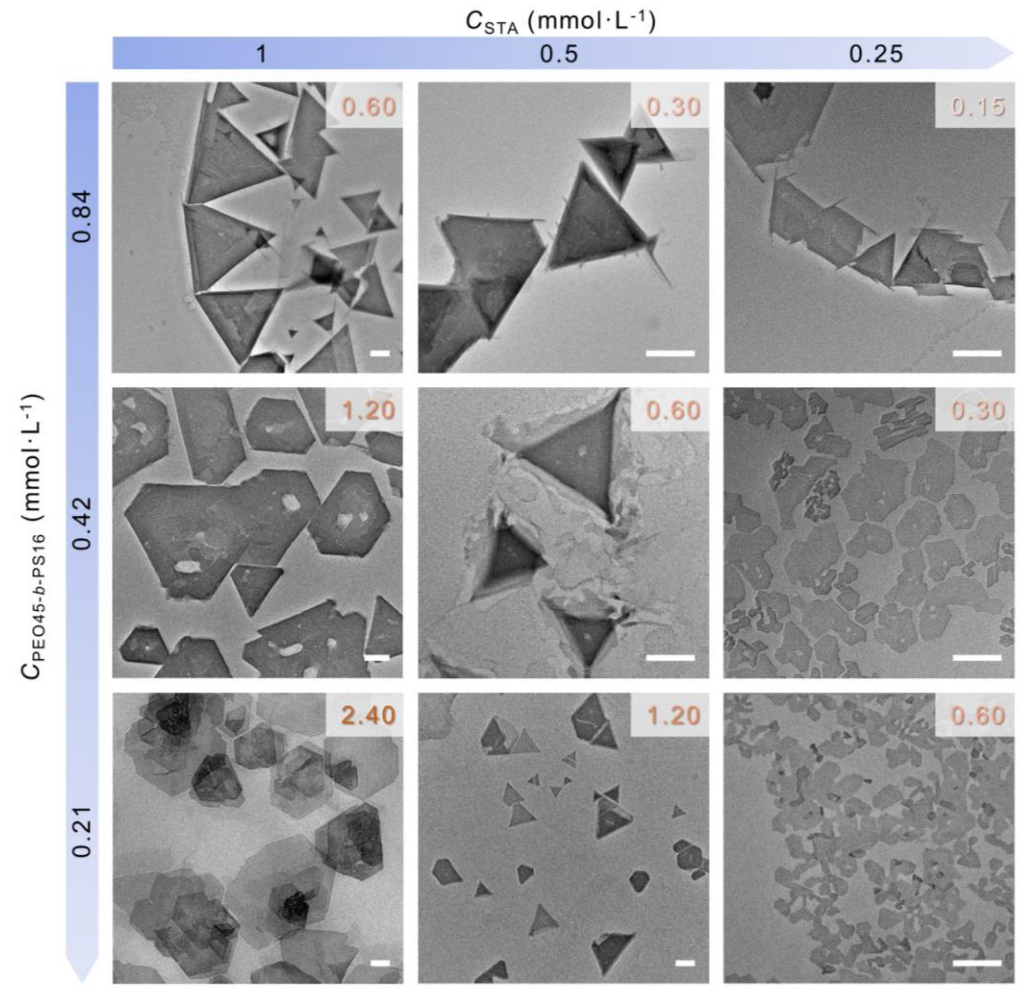


**Supplementary Fig. 12 | TEM images of STA/$PEO_{45}$/$PEO_{45}$-*b*-$PS_{16}$ at different concentrations and molar ratios of STA to total PEO at the fixed $\varphi_{PEO\text{-}b\text{-}PS}$ of 50%.** The corresponding molar ratio values, *n*(STA)/*n*($PEO_{45}$ + $PEO_{45}$-*b*-$PS_{16}$), were marked in the upper left corner of the images. All scale bars are fixed to 200 nm.

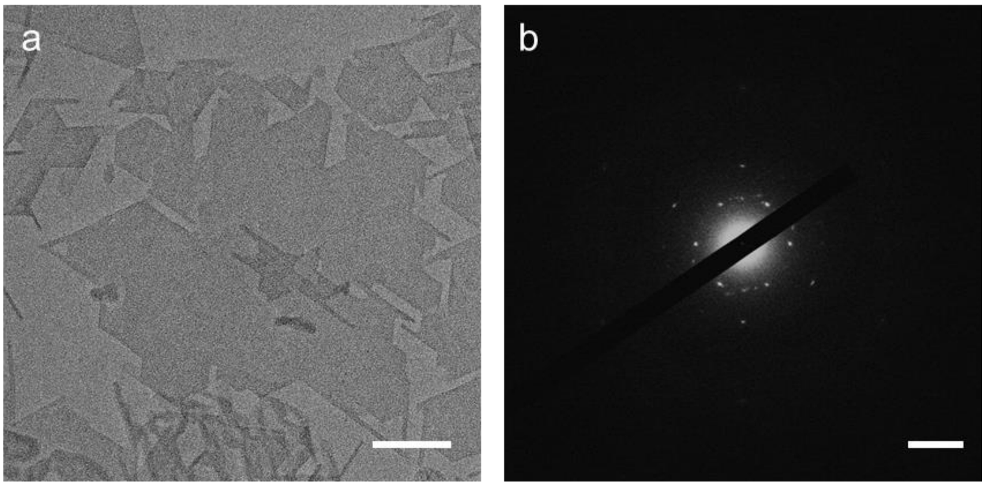


**Supplementary Fig. 13 | Co-crystallization-driven self-assembly by $PEO_{113}$-*b*-$PS_{30}$ and STA.** TEM image (**a**) and SAED pattern (**b**) of the triangular nanoplates assembled from $PEO_{113}$-*b*-$PS_{30}$ and STA. The scale bars are 100 nm for (**a**) and 1 $nm^{-1}$ for (**b**).

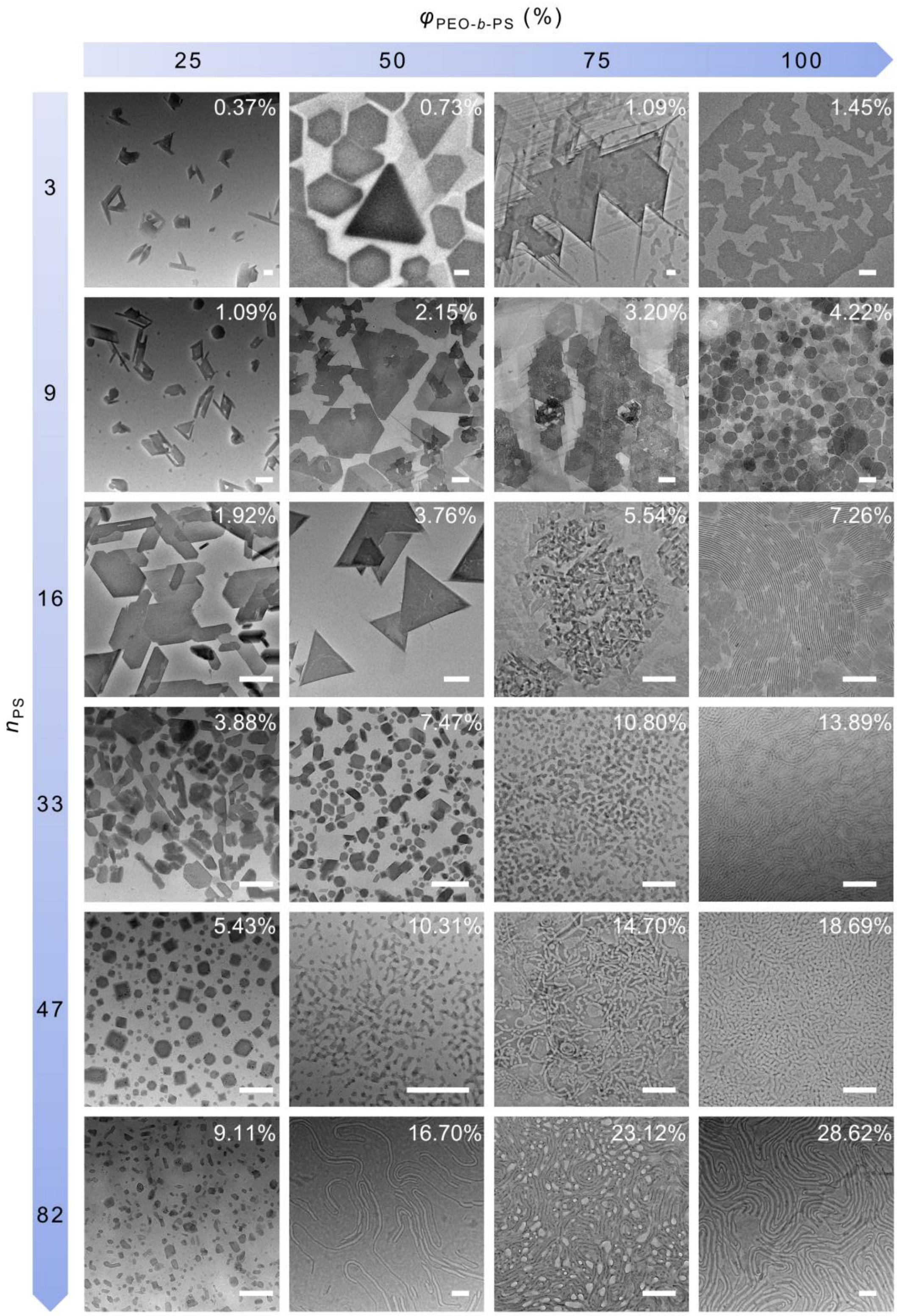


**Supplementary Fig. 14 | TEM images of ternary assemblies of STA/PEO/$PEO_{45}$-*b*-$PS_n$ with various volume fractions of PS ($f_{PS}$).** The corresponding $f_{PS}$ is marked in the upper left corner of the picture. All scale bars are fixed to 100 nm.

# 4. Reference


1. Petříček, V., Dušek, M. & Palatinus, L. Crystallographic Computing System JANA2006: General features. *Zeitschrift für Kristallographie - Crystalline Materials* **229**, 345-352 (2014).
2. Hansen, C. M. *Hansen Solubility Parameters: A Users Handbook, Second Edition* (CRC Press, Boca Raton, 2007).
